# Optimal Seasonal Timing of Oral Azithromycin for Malaria

**DOI:** 10.4269/ajtmh.13-0474

**Published:** 2014-11-05

**Authors:** Daozhou Gao, Abdou Amza, Baidou Nassirou, Boubacar Kadri, Nicholas Sippl-Swezey, Fengchen Liu, Sarah F. Ackley, Thomas M. Lietman, Travis C. Porco

**Affiliations:** F.I. Proctor Foundation for Ophthalmological Research, University of California, San Francisco, California; Université Abdou Moumouni de Niamey, Programme National de Lutte Contre la Cécité, Niamey, Niger; Department of Ophthalmology, Department of Epidemiology and Biostatistics, University of California, San Francisco, California; Institute for Global Health, University of California, San Francisco, California

## Abstract

Mass administration of azithromycin for trachoma has been shown to reduce malarial parasitemia. However, the optimal seasonal timing of such distributions for antimalarial benefit has not been established. We performed numerical analyses on a seasonally forced epidemic model (of Ross-Macdonald type) with periodic impulsive annual mass treatment to address this question. We conclude that when azithromycin-based trachoma elimination programs occur in regions of seasonal malaria transmission, such as Niger, the optimal seasonal timing of mass drug administration (MDA) may not occur during the season of maximum transmission.

## Introduction

Malaria is a leading cause of morbidity and mortality among young children in sub-Saharan Africa.^1^ Current World Health Organization (WHO) recommendations for malaria control include insecticide-treated nets, indoor residual spraying, and seasonal malaria chemoprevention (administering antimalarial treatment during the period of greatest risk to prevent illness).^2^ Current recommendations indicate the intermittent use of a full course of amodiaquine and sulfadoxine-pyrimethamine initiated at the beginning of the peak transmission season.^2^

However, other drugs may also have a role in eliminating malaria. Mass distribution of azithromycin, a second choice for antimalarial therapy in multidrug-resistant or chloroquine-resistant areas,^3^,^4^ is a cornerstone of the WHO trachoma elimination program, dramatically reducing the prevalence of the ocular strains of *Chlamydia* that cause trachoma.^5^,^6^ Such efforts may reduce malaria transmission as well, because of the antimalarial effects of azithromycin.^3^,^4^

Previous models have addressed the question of seasonally timed intermittent preventive therapy (IPT), finding that IPT in children (IPTc) has a significant potential to reduce malaria transmission, particularly in low to moderate transmission areas.^7^ The IPT is typically implemented at the beginning of or during the time of highest transmission. However, mass administration of azithromycin is not designed to prevent infection, but to eliminate established infection. We do not expect the optimal seasonal timing to be the same as for IPT.

Should mass administration of azithromycin for trachoma be timed during the peak season of malaria transmission if we wish to obtain maximum antimalarial benefit as well? Treatment during a time of lower transmission may be more promising, yielding durable cures because of the low risk of reinfection at such time, and furthermore, lowering the number of infectious individuals at the beginning of the next peak season. To determine the optimal seasonal timing of mass treatment with azithromycin, we used a simple mathematical model similar to the classical model of Ross and Macdonald^8^,^9^ to incorporate a seasonally varying mosquito abundance^10^–^12^ in a small community setting.

## Materials and Methods

### Mathematical model.

We examined the transmission dynamics of malaria using a simple mathematical model of Ross-Macdonald type.^8^,^9^ For simplicity, we divided the human population into two age classes, children < 12 years of age, and everyone else. We assume a fixed human population size. In malaria-endemic areas, parasitemia levels in adult humans are typically much lower than in children, and the gametocytes in individuals with acquired partial immunity have low infectivity to mosquitoes,^13^,^14^ and so the contribution of adult humans to malaria transmission is omitted.

Any child < 12 years of age may be susceptible or infected. Each mosquito bites humans at a given rate per unit time, and if the mosquito is infectious, transmits infection to susceptible children with some specified probability. Infected children are assumed to recover at a given rate, becoming susceptible once again. Similarly, mosquitoes are assumed to be either susceptible or infectious. An uninfected (susceptible) mosquito becomes infected when it feeds from infected children and ingests gametocytes from the blood of that person. Adult mosquitoes are assumed to have a mean lifetime of a few days to several weeks. The contribution of adults^15^ and the exposed period in mosquitoes^16^–^18^ are considered in the Supplemental Material.

As we know, seasonal changes in mosquito abundance could lead to periodic outbreaks of malaria.^19^ The mosquito population as a whole is assumed to follow a specified seasonally varying function with large changes in mosquito abundance over the course of 1 year. In Niger, the peak mosquito abundance typically occurs from early August to late October, lasting 6 weeks to 3 months.^20^ Mathematically, we represent this variation by assuming that the vector abundance varies between a small value during the low abundance season and a large value during the high abundance season, using a simple periodic function described in the Appendix. For convenience, we divide the year into four regions: the time during which mosquito abundance is (arbitrarily) greater than the cutoff value 5% of the peak value (the peak mosquito season or high abundance season), 1 month before this peak season, 1 month after this peak season, and finally, the rest of the year (the season of low mosquito abundance).

Annual treatment is modeled by moving a fraction of infectives to the susceptible class after a single distribution of oral azithromycin. The recommended antibiotic coverage for trachoma control by WHO is at least 80% and the daily azithromycin intakes show high protective efficacy against *Plasmodium vivax* malaria of 98.9%; however, modest efficacy against *Plasmodium falciparum* malaria of 71.6%.^3^,^4^,^21^ The major *Plasmodium* species in Niger is *P. falciparum*.

Finally, we assumed a large fraction of the human population was < 12 years of age. Model parameters are shown in Table 1, together with an uncertainty distribution for each.

### Numerical simulations.

We determined the effect of timing of mass administration of azithromycin on malaria incidence and prevalence by numerical simulations. For some parameter sets, repeated impulsive mass treatment at a time or any time is sufficient to eliminate malaria entirely. For other parameter sets, annual mass treatment leads to a stable periodic solution; malaria levels change over the course of each year, but repeat the same values annually. This implies that the same level of malaria prevalence is achieved at the beginning of each season. Given all other model parameters (e.g., peak mosquito abundance, biting rate), we determined a long-run equilibrium value for the prevalence in humans and mosquitoes during the beginning of the year, by simulating many years of transmission and treatment.

We considered two measures of public health benefit for any parameter set. First, we computed the total person-time of infection per year, denoted by *P*, using this long run solution for the epidemic. Second, we computed the total annual incidence of infection (number of new infections over a year), denoted by *Q*.

When all other parameters are fixed, the choice of treatment time, τ, changes, in general, the total person-time and the total annual incidence. We computed the times 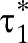 and 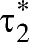 for which the person-time and the total annual incidence were minimized, respectively. Unless otherwise stated, the optimal treatment time throughout this work is the former.

In addition to the time at which the total person-time infected was minimized, we also found the worst time of year for treatment. We determined the difference in person-time between the best time and the worst time to treat, providing a measure of the importance of seasonal timing of mass administration.

For simulation, we let the mosquito abundance attain its peak in the middle of August, corresponding approximately to Niger. Each parameter set yields an optimal time of treatment (or else yields malaria extinction for some treatment times).

For each scenario, we also computed three additional quantities over the season of peak mosquito abundance: 1) the entomological inoculation rate (EIR), 2) the cumulative hazard of infection (CHI), and 3) the basic reproduction number.

Sensitivity analysis for parameters influencing the optimal treatment time was conducted by computing the circular-linear partial rank correlation coefficient (CLPRCC) between the optimal treatment time and each parameter. Because the optimal treatment time is a point in a cycle, times near the end of the year are close to times near the beginning of the year, necessitating the use of circular statistics. The CLPRCC is computed by replacing linear variables by their ranks and angular variables by their circular ranks,^29^,^30^ and computing the circular-linear partial correlation coefficient (see Appendix for details). For sensitivity analysis of factors influencing the difference in person-time infected between the best and worst times to treat, we used the usual partial rank correlation coefficient.

### Data collection.

As a guide to what the model results may mean in practice, we compared model results to data from the Partnership for the Rapid Elimination of Trachoma (PRET) study, Niger arm. The PRET trial is a multicenter community randomized trachoma control trial, with study sites in Tanzania, the Gambia, and Niger^31^,^32^; for this study, we used malaria outcome data obtained in the Niger site. In brief, in Niger, 48 communities (*grappes*) were randomized (using a 2 × 2 factorial design) to one of four treatment assignments based on enhanced versus standard azithromycin coverage, and annual mass treatment of the entire community versus twice-yearly treatment of only children. Communities were selected from among six health centers in the Matameye district (Zinder region).

Stratified randomization was conducted by 1) obtaining baseline measurements of follicular trachoma by field examination, 2) classifying the communities within each health district into the upper half or lower half of trachoma prevalence, and 3) choosing eight (two each for each of four arms) from each health center such that one of the assignments to each arm would always be in the lowest half of baseline trachoma prevalence.

The malaria collection and results have been described elsewhere^33^; in brief, thick blood smears and blood spots were obtained from communities in standard assignment, both for villages assigned to the annual mass treatment and for villages assigned to twice-yearly treatment of only children. These specimens were collected post treatment, at the 1 year time (January 2011), from a census-based cross-sectional random sample of 50 children (or all children, if fewer than 50 were present in the village). Thick blood smears were examined under light microscopy after 3% Giemsa staining by two graders.

### Model analysis.

The previous mathematical model contains parameters for mosquito abundance, transmission rate, and mosquito lifetime, which are not specific to any region, and may range over values corresponding to hypoendemic, mesoendemic, and hyperendemic settings. Is it possible that parameter values corresponding to field settings where malaria is highly seasonal yield different results than other settings? We examined data from the annual arm of the PRET study, comparing the 10% of the scenarios that were closest to the observed data to the other 90%. For this mathematical modeling study, we computed, for each village, the mean prevalence of parasitemia assessed as positive by either of the two graders, yielding 12 community-specific prevalences 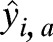 (i = 1,…,12) for the annually treated communities. For each parameter set, the mathematical model was simulated until it reached its stable cyclic value. For each annually treated village, we simulated a treatment at the beginning of the study. At this time, we computed the product of the coverage for that village and the assumed efficacy of the antibiotic in clearing malaria, and assumed that fraction of children were cured; the remaining children stayed infected. We simulated that village until the data collection time. Finally, we computed the absolute value of the difference between the modeled value for the prevalence and the actual prevalence and determined the best treatment time.

## Results

The malaria model with treatment shows that mass azithromycin distribution could reduce malaria transmission, and may even eliminate the disease in low transmission settings. Of course, this finding depends on the assumption of moderate to high efficacy of azithromycin.

**Figure 1.**
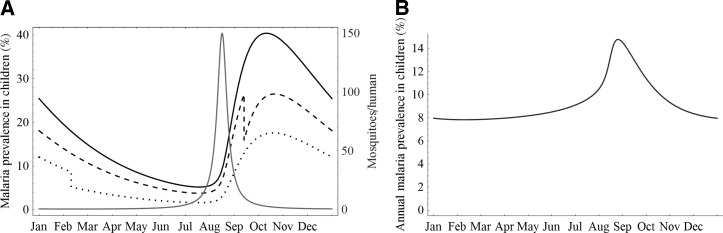
Simulated malaria prevalence in children and the ratio of mosquitoes to humans versus time. The parameters are as follows: *a* = 4, *b* = 0.33, *c* = 0.16, *r* = 0.33, μ = 1.205, σ = 0.325, *H* = 580, *m* = 3.15, *k* = 0.9917, *p* = 0.5, and *q* = 0.8. (**A**) Solid black line-prevalence curve given no mass azithromycin treatment, dashed line-prevalence curve given a suboptimal treatment time in September, dotted line-prevalence curve given treatment at optimal time in February, gray solid line-mosquito to human ratio; (**B**) annual malaria prevalence in children varies with respect to initial treatment time.

When we assume that the mosquito population is constant, it makes no difference when an annual treatment occurs. Similarly, if the mosquito population is assumed to undergo only small fluctuations, timing of mass administration makes little difference. However, in cases of pronounced seasonality, as may occur in Niger and other parts of the Sahel, timing of mass administration has a larger effect, as seen in Figure 1. In Figure 1A, we present the prevalence of malaria in children over the course of 1 year (at equilibrium), assuming no treatment, and then assuming treatment at a suboptimal time and at the optimal time. In the absence of mass antibiotic administration, malaria prevalence (black solid line) fluctuates over the year and attains its maximum 2 months after mosquito high abundance season (gray curve). Treatment may be administered at any time but can have quite different effects. The prevalence in young humans is largely reduced when azithromycin is distributed in the low abundance season (dotted line), but only slightly reduced when mass administration occurs in the season of high mosquito abundance (dashed line). Treatment during the season of peak mosquito abundance is less effective, because individuals are quickly reinfected. For the same parameter sets, the relation between malaria prevalence in children and its antibiotic treatment time is clearly shown in Figure 1B.

**Figure 2.**
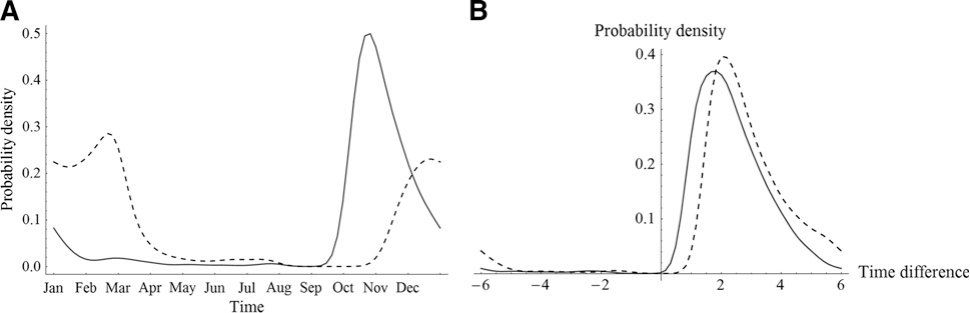
(**A**) Smoothed probability density plot of optimal treatment times with respect to annual prevalence (solid line) and incidence (dashed line), respectively. (**B**) Smoothed probability density plot of the circular differences between the optimal time with respect to annual prevalence and the peak point prevalence time (solid line), and the circular differences between the optimal time with respect to annual prevalence and the peak point incidence time (dashed line). Here, the baseline range of parameter values can be found in Table 1.

To determine when it is best to give a single dose of azithromycin to everyone in the population, we first chose plausible input ranges for model parameters, generated 10,000 uniformly distributed random parameter sets, and then computed the optimal treatment times to minimize the equilibrium person-time spent and the annual incidence of infection, respectively, and evaluated the difference in person-years between the best and worst times to treat, given each set. When the disease goes extinct under treatment at a time or any time, we exclude the corresponding parameter set. For each qualified simulation (where the disease remains persistent after treatment any time) we find that there always exists a unique optimal treatment time. Figure 2A shows two smoothed density plots of optimal times in terms of annual prevalence and incidence, respectively. The mosquito abundance reaches its maximum in the middle of August, which usually precedes the optimal treatment times.

Among these sets, 30 occurred during the season of highest mosquito abundance, 5,542 occurred during times of the year when mosquitoes were not abundant (10, 1 month before the peak season; 1,365, 1 month after the peak season; 4,167 during the season of low mosquito abundance); and the disease disappears without treatment or with treatment at a time or any time in the remaining 4,428 cases. Of our scenarios, 4,194 corresponded to parameter choices for which malaria cannot remain endemic even without treatment because of the basic reproduction number *R*_0_ for the corresponding model without treatment satisfying *R*_0_ < 1, and therefore contribute no further to the analysis.^12^ Averaging over all non-eradication scenarios, the minimum malaria prevalence attained when the optimal time is chosen is 12.3% lower than that seen if the mass treatment occurs during the worst time.

The distribution of the circular differences between the optimal treatment time and the peak point prevalence time, and the circular differences between the optimal treatment time and the peak point incidence time, are plotted in Figure 2B. On average, the optimal treatment time is 2 to 3 months after the peak point prevalence time and peak point incidence time. Therefore, for seasonal malaria transmission, it is usually better to conduct mass administration in the mosquito low abundance season or after the high abundance season.

We computed the CHI and EIR over the season of peak mosquito abundance in the absence of treatment. For the parameter sets for which elimination does not occur, the average CHI and EIR for scenarios yielding an optimal treatment time in the high abundance season are much smaller than those in the low abundance season, and the ratios are 1:23.3 and 1:16.5, respectively. The average minimum annual prevalence for scenarios yielding an optimal treatment time in the high abundance season and low abundance seasons are 8.8% and 31.5%, respectively. We also computed the basic reproduction number *R_h_*, which would be obtained if the high mosquito abundance were sustained, and found that the average *R_h_* for scenarios yielding an optimal treatment time at high abundance season and low abundance season are 3.1 and 5.9, respectively. Scenarios in which the optimal treatment time occurs during the peak mosquito abundance season correspond to less transmission. The same conclusions still apply when we choose a different cutoff value for mosquito high/low abundance season ranging from 3% to 15% (see Supplemental Material for details).

In addition, we report the CLPRCC for sensitivity analysis (see Table 2). The distribution of optimal times, and the difference in person-time between the best and worst treatment times are most sensitive to the mortality rate of mosquitoes and the recovery rate of children, respectively.

If we restrict our attention to parameter sets yielding the least discrepancy when compared to the cross-sectional community prevalence data from the PRET study in Niger, what is the best time to treat? Restricting ourselves to the best 10% of parameter sets, the circular mean of the best time corresponds to approximately November 20, with plus or minus one circular standard deviation corresponding to the period from November 7 to December 4.

The extrinsic incubation period in mosquitoes has a large impact on malaria transmission because most infected mosquitoes will die before they become infectious.^16^–^18^ In some areas adults are responsible for a large proportion of the infectious reservoir to mosquitoes.^15^ To take these into consideration, we developed an age-structured model with an SEI (susceptible-exposed-infectious) pattern for mosquitoes^34^ and found that the central tendency of optimal treatment times is essentially unchanged (see Supplemental Material).

## Discussion

A simple Ross-Macdonald malaria model with seasonally varying mosquito density permitted an assessment of optimal time of year for mass treatment using oral azithromycin. For minimizing the total person-time of infection, the optimal time occurs after peak transmission. When the entomological inoculation rate is high, mass treatment of humans with azithromycin may be largely futile, because individuals are rapidly reinfected. Infected mosquitoes remain infectious after the mass treatment, and in any case rapid amplification of infection in the human population during the mosquito season attenuates the benefit of mass treatment of humans at a single time. Treatment during the transmission peak may be optimal in the low transmission setting.

Our analysis does not inform seasonal malaria chemoprevention, which is designed to prevent infection. The WHO has recommended IPTc for the control of malaria in the Sahel during the wet, high-transmission season in areas with highly seasonal malaria transmission such as the Sahel.^35^–^39^

Simple models of Ross-MacDonald form capture important relationships between mosquito abundance, biting rates, and transmission, but do not purport to represent detailed vector ecology and biogeography. Such model may be extended to include substantial fluctuations in vector abundance, therefore the optimal timing could be customized to local conditions. Although the finding that a mass treatment is ideally timed after peak transmission, under conditions when a low abundance of the vector occurs, the timing could be moved to the transmission season. Similarly, the effect of bed nets and other control measures, when successful, could affect the optimal seasonal timing. Extension of our model to include additional features of the malaria transmission system, such as infection-dependent mortality and the duration of antibiotic efficacy may be useful in further assessing the role that mass distribution of azithromycin may play in malaria amelioration. The dynamics of malaria in areas of low transmission may be driven by imported infections that can have a different seasonal pattern than that of the mosquito population.

Two other investigations have suggested that treatment during the low transmission season of an infectious disease may be beneficial,^40^,^41^ and hypothesized that this may be because circulating malarial parasites eliminated with treatment are not replaced in the low transmission setting.^40^ Mass drug administration before the malaria transmission season may prevent the parasite prevalence levels from recovering to their pretreatment levels, and may even allow parasite elimination in these low transmission settings.^41^ Mathematical models of mass treatment of trachoma have found that the optimum treatment time to achieve elimination is during the season of lowest transmission.^42^ Thus, seasonal variations in transmission can be exploited to maximize the impact of mass drug administration.^41^ Depending on the transmission dynamics, the optimal timing may occur away from the time of maximum transmission.

## Supplementary Material

Supplemental File.

## Figures and Tables

**Table 1:** Parameters of the malaria model with description, range, baseline, unit, and reference

	Description	Range	Baseline	Unit	References
*a*	The number of bites per mosquito per month	3–30	3–15	Bites per mosquito per month	18,22–24
*b*	Transmission probability from infected mosquitoes to susceptible children per bite	0.01–0.8	0.1–0.5	Per bite	18,22,25–27
*c*	Transmission probability from infected children to susceptible mosquitoes per bite	0.072–0.64	0.1–0.5	Per bite	23,25–27
1 / *r*	Duration of infectiousness	0.7–10	0.7–3	Month	18,24–27
1 / μ	Lifespan of mosquitoes	0.2–36	0.25–1	Month	23–25
σ	The proportion of people under 12	0–1	0.25–0.5	−	28
*H*	Number of humans	50–1000	250–600	−	Assume
*m*	Average ratio of mosquitoes to humans	1–10	1–4	Mosquitoes per human	18,22–24
*k*	Measure of the duration of high abundance season	0–1	0.984–0.997	−	20
*p*	Curative efficacy of single dose of azithromycin	0–1	0.4–0.8	−	Assume
*q_i_*	Treatment coverage	0–1	0.6–0.9	−	Assume
τ_1_	Initial mass administration time	0–1	0–1	Year	Assume

**Table 2:** Sensitivity analysis of model outcomes with respect to model parameters*

	Parameter description	PRCC of the difference in annual person-time of infection comparing best and worst	CLPRCC for the optimal treatment time for minimizing annual person-time
*a*	Mosquito biting rate	0.09	0.33
*b*	Transmission probability from mosquitoes to humans	0.17	0.35
*c*	Transmission probability from humans to mosquitoes	−0.01	0.18
*r*	Recovery rate of children	−0.97	0.34
μ	Mortality rate of mosquitoes	0.62	0.59
σ	Proportion of children	−0.02	0.11
*H*	Total number of humans	−0.01	0.03
*m*	Ratio of mosquitoes to humans	0.16	0.29
*k*	Measure of the duration of high abundance season	0.08	0.19
*p*	Curative efficacy of single dose of azithromycin	0.84	0.04
*q_i_* = *q*	Treatment coverage	0.66	0.01

* Circular-linear partial rank correlation coefficient (CLPRCC) for optimal treatment time (for minimizing annual person-time); values of the CLPRCC vary between 0 (little association) and 1 (strong association). Linear partial rank correlation coefficient (PRCC) for the difference in person-time of infection between best and worst times of the year to treat.

## APPENDIX

The malaria transmission model is as follows. Because the malaria parasite density and the prevalence of gametocytemia are small in adulthood,^14^ we only model transmission among children < 12 years of age. Let *V*(*t*) and *H* be the total number of mosquitoes and humans, respectively. A constant fraction σ of humans is < 12 years of age. Let *v*(*t*) and *h*(*t*) be the population size of infected mosquitoes and infected children, respectively. Consider a simple model of Ross-Macdonald type with seasonally forced vector abundance and periodic impulsive annual mass treatment:

where  is the left-hand limit of *h*(*t*) at *t* = τ*_i_*, δ(*t*) is the Dirac delta function, and  satisfies 0 ≤ τ_1_ < 1 and τ*_i_*_+1_ = τ*_i_* + 1. We omit human vital dynamics and migration.

Here, the parameter *a* is the number of humans a mosquito bites per year, *b* is the probability of transmission of infection to susceptible children by an infected mosquito, *c* is the probability an infected child infects a susceptible mosquito, *r* is the recovery rate for children, μ is the death rate for mosquitoes. The *q_i_* is treatment coverage at time τ*_i_* for children, and *p* is the efficacy of antibiotic treatment in children (i.e., the probability of cure after treatment). All time independent parameters are positive constants, and *V*(*t*) is a positive, continuous, and periodic function with period 1.

Some authors have found that the model (1) without treatment, while simple, is mathematically and epidemiologically reasonable for obtaining insight into malaria dynamics.^43^ In this work, we chose the following periodic form for mosquito density function

where *m* > 0 is the average ratio of mosquitoes to humans, Λ(*t*) describes the seasonal changes,^11^ and 0 < *k* < 1 is a coefficient that controls the duration of high abundance season. For this function, the mosquito abundance reaches its maximum in the middle of August (*t* = 5 / 8, 1 + 5 / 8, 2 + 5 / 8, …). The larger the value of *k*, the shorter the length of the high abundance season and the larger the range over which the mosquito density varies.

Define the person-time of infection per year, *P*, annual incidence of infection, *Q*, the cumulative hazard, *CHI*, and the entomological inoculation rate, *EIR,* for one season at equilibrium as the definite integral of the proportion of infected children, number of new infections over 1 year, the force of infection, and the number of bites by infectious mosquitoes per person per year from *t_A_*, the beginning of the high abundance season, to *t_B_*, the end of the high abundance season, respectively. In other words, *P* = *X*(1), *Q* = *Y*(1), *CHI* = *Z*(*t_B_*), and *EIR* = *W*(*t_B_*), where *X*(*t*), *Y*(*t*), *Z*(*t*), and *W*(*t*) are the equilibrium solution of the following initial value problems

together with Equation (1) for *q_i_* = 0 and

together with Equation (1) for *q_i_* = 0, respectively. Specifically, the optimal treatment times in terms of annual prevalence and incidence are  and , respectively.

The basic reproduction number *R_h_* is defined for the following periodic model^44^,^45^

over high mosquito abundance season [*t_A_*, *t_B_*].

The circular-linear correlation *r* between an angular variable θ and a linear variable *x* is computed from

where *r*_12_ is the Pearson correlation between cos θ and sin θ, *r*_13_ is the Pearson correlation between cos θ and *x*, and *r*_23_ the Pearson correlation between sin θ and *x*. We computed the circular-linear partial rank correlation of θ and any variable *x* with the others held constant by using the previous formula, replacing linear quantities by their ranks, circular quantities by their circular ranks, and computing the correlations from the residuals of cos θ, sin θ, and *x* (any parameter of interest) after regression on all other parameters of interest (exactly analogous to computation of partial correlation coefficients that do not involve angular variables). The previous circular-linear correlation coefficient ranges from 0 (no association) to 1 (perfect association), and measures the strength of the association between the linear variable *x* and any function *A* cos (θ + φ) where *A* is any constant, and φ is any arbitrary phase.

Numerical analysis was conducted using Mathematica 9.0 for finding the optimal time. Differential equations were numerically solved using NDSolve with option LSODA, and random numbers were generated by the default method in Mathematica 9.0 (a cellular automaton-based method^46^).

## References

[1] WHO (2012). World Malaria Report 2012.

[2] WHO (2012). WHO policy recommendation: seasonal malaria chemoprevention (SMC) for *Plasmodium falciparum* malaria control in highly seasonal transmission areas of the Sahel sub-region in Africa.

[3] Taylor WR, Richie TL, Fryauff DJ, Picarima H, Ohrt C, Tang D, Braitman D, Murphy GS, Widjaja H, Tjitra E, Ganjar A, Jones TR, Basri H, Berman J (1999). Malaria prophylaxis using azithromycin: a double-blind, placebo-controlled trial in Irian Jaya, Indonesia. Clin Infect Dis.

[4] van Eijk AM, Terlouw DJ (2011). Azithromycin for treating uncomplicated malaria. Cochrane Database Syst Rev.

[5] Melese M, Chidambaram JD, Alemayehu W, Lee DC, Yi EH, Cevallos V, Zhou Z, Donnellan C, Saidel M, Whitcher JP, Gaynor BD, Lietman TM (2004). Feasibility of eliminating ocular *Chlamydia trachomatis* with repeat mass antibiotic treatments. JAMA.

[6] Schachter J, West SK, Mabey D, Dawson CR, Bobo L, Bailey R, Vitale S, Quinn TC, Sheta A, Sallam S, Mkocha H, Mabey D, Faal H (1999). Azithromycin in control of trachoma. Lancet.

[7] Aguas R, Lourenco JM, Gomes MG, White LJ (2009). The impact of IPTi and IPTc interventions on malaria clinical burden - *in silico* perspectives. PLoS ONE.

[8] Ross SR (1910). The Prevention of Malaria.

[9] Macdonald G (1952). The analysis of equilibrium in malaria. Trop Dis Bull.

[10] Smith DL, Dushoff J, McKenzie FE (2004). The risk of a mosquito-borne infection in a heterogeneous environment. PLoS Biol.

[11] Wyse AP, Bevilacqua L, Rafikou M (2007). Simulating malaria model for different treatment intensities in a variable environment. Ecol Modell.

[12] Gao D, Lou Y, Ruan S (2014). A periodic Ross-Macdonald model in a patchy environment. Discrete and Continuous Dynamical Systems-Series B.

[13] Mayor A, Aponte JJ, Fogg C, Saute F, Greenwood B, Dgedge M, Menendez C, Alonso PL (2007). The epidemiology of malaria in adults in a rural area of southern Mozambique. Malar J.

[14] Anderson RM, May RM (1991). Infectious Diseases of Humans: Dynamics and Control.

[15] Ross A, Killeen G, Smith T (2006). Relationships between host infectivity to mosquitoes and asexual parasite density in *Plasmodium falciparum*. Am J Trop Med Hyg.

[16] Smith DL, McKenzie FE (2004). Statics and dynamics of malaria infection in *Anopheles* mosquitoes. Malar J.

[17] Smith DL, Battle KE, Hay SI, Barker CM, Scott TW, McKenzie FE (2012). Ross, Macdonald, and a theory for the dynamics and control of mosquito-transmitted pathogens. PLoS Pathog.

[18] Ruan S, Xiao D, Beier JC (2008). On the delayed Ross-Macdonald model for malaria transmission. Bull Math Biol.

[19] Grassly NC, Fraser C (2006). Seasonal infectious disease epidemiology. Proc Biol Sci.

[20] Bomblies A, Duchemin JB, Eltahir EA (2008). Hydrology of malaria: model development and application to a Sahelian village. Water Resour Res.

[21] Dunne MW, Singh N, Shukla M, Valecha N, Bhattacharyya P, Dev V, Patel K, Mohapatra MK, Lakhani J, Benner R (2005). A multicenter study of azithromycin, alone and in combination with chloroquine, for the treatment of acute uncomplicated *Plasmodium falciparum* malaria in India. J Infect Dis.

[22] Eckhoff PA (2011). A malaria transmission-directed model of mosquito life cycle and ecology. Malar J.

[23] Killeen GF, McKenzie FE, Foy BD, Schieffelin C, Billingsley PF, Beier JC (2000). A simplified model for predicting malaria entomologic inoculation rates based on entomologic and parasitologic parameters relevant to control. Am J Trop Med Hyg.

[24] Edlund S, Davis M, Douglas JV, Kershenbaum A, Waraporn N, Lessler J, Kaufman JH (2012). A global model of malaria climate sensitivity: comparing malaria response to historic climate data based on simulation and officially reported malaria incidence. Malar J.

[25] Chitnis N, Hyman JM, Cushing JM (2008). Determining important parameters in the spread of malaria through the sensitivity analysis of a mathematical model. Bull Math Biol.

[26] Ermert V, Fink AH, Jones AE, Morse AP (2011). Development of a new version of the Liverpool Malaria Model. I. Refining the parameter settings and mathematical formulation of basic processes based on a literature review. Malar J.

[27] Ermert V, Fink AH, Jones AE, Morse AP (2011). Development of a new version of the Liverpool Malaria Model. II. Calibration and validation for West Africa. Malar J.

[28] INS-Niger (2011). Le Niger en Chiffres 2011. http://www.stat-niger.org/statistique/file/Annuaires_Statistiques/Annuaire_ins_2011/Niger%20en%20chiffres%20nov%202011.pdf.

[29] Fisher NI (1993). Statistical Analysis of Circular Data.

[30] Johnson RA, Wehrly T (1977). Measures and models for angular-correlation and angular-linear correlation. J R Stat Soc, B.

[31] Stare D, Harding-Esch E, Munoz B, Bailey R, Mabey D, Holland M, Gaydos C, West S (2011). Design and baseline data of a randomized trial to evaluate coverage and frequency of mass treatment with azithromycin: the Partnership for Rapid Elimination of Trachoma (PRET) in Tanzania and The Gambia. Ophthalmic Epidemiol.

[32] Amza A, Kadri B, Nassirou B, Stoller NE, Yu SN, Zhou Z, Chin S, West SK, Bailey RL, Mabey DC, Keenan JD, Porco TC, Lietman TM, Gaynor BD, Partnership P (2012). Community risk factors for ocular *Chlamydia* infection in Niger: pre-treatment results from a cluster-randomized trachoma trial. PLoS Negl Trop Dis.

[33] Gaynor BD, Amza A, Kadri B, Nassirou B, Lawan O, Maman L, Stoller NE, Yu SN, Zhou Z, Chin S, West SK, Bailey RL, Rosenthal PJ, Keenan JD, Porco TC, Lietman TM (2014). Impact of mass azithromycin distribution on malarial parasitemia during the low-transmission season in Niger: a cluster-randomized trial. Am J Trop Med Hyg.

[34] Gao D, Ruan S (2012). A multi-patch malaria model with logistic growth populations. SIAM J Appl Math.

[35] Cisse B, Sokhna C, Boulanger D, Milet J, Ba el H, Richardson K, Hallett R, Sutherland C, Simondon K, Simondon F, Alexander N, Gaye O, Targett G, Lines J, Greenwood B, Trape JF (2006). Seasonal intermittent preventive treatment with artesunate and sulfadoxine-pyrimethamine for prevention of malaria in Senegalese children: a randomized, placebo-controlled, double-blind trial. Lancet.

[36] Konate AT, Yaro JB, Ouedraogo AZ, Diarra A, Gansane A, Soulama I, Kangoye DT, Kabore Y, Ouedraogo E, Ouedraogo A, Tiono AB, Ouedraogo IN, Chandramohan D, Cousens S, Milligan PJ, Sirima SB, Greenwood B, Diallo DA (2011). Intermittent preventive treatment of malaria provides substantial protection against malaria in children already protected by an insecticide-treated bed net in Burkina Faso: a randomized, double-blind, placebo-controlled trial. PLoS Med.

[37] Near KA, Stowers AW, Jankovic D, Kaslow DC (2002). Improved immunogenicity and efficacy of the recombinant 19-kilodalton merozoite surface protein 1 by the addition of oligodeoxynucleotide and aluminum hydroxide gel in a murine malaria vaccine model. Infect Immun.

[38] Daubenberger CA, Salomon M, Vecino W, Hubner B, Troll H, Rodriques R, Patarroyo ME, Pluschke G (2001). Functional and structural similarity of V gamma 9V delta 2 T cells in humans and *Aotus* monkeys, a primate infection model for *Plasmodium falciparum* malaria. J Immunol.

[39] Wilson AL, Taskforce IP (2011). A systematic review and meta-analysis of the efficacy and safety of intermittent preventive treatment of malaria in children (IPTc). PLoS ONE.

[40] Gu W, Killeen GF, Mbogo CM, Regens JL, Githure JI, Beier JC (2003). An individual-based model of *Plasmodium falciparum* malaria transmission on the coast of Kenya. Trans R Soc Trop Med Hyg.

[41] Okell LC, Griffin JT, Kleinschmidt I, Hollingsworth TD, Churcher TS, White MJ, Bousema T, Drakeley CJ, Ghani AC (2011). The potential contribution of mass treatment to the control of *Plasmodium falciparum* malaria. PLoS ONE.

[42] Lee DC, Chidambaram JD, Porco TC, Lietman TM (2005). Seasonal effects in the elimination of trachoma. Am J Trop Med Hyg.

[43] Lou Y, Zhao X-Q (2010). The periodic Ross-Macdonald model with diffusion and advection. Appl Anal.

[44] Bacaër N, Guernaoui S (2006). The epidemic threshold of vector-borne diseases with seasonality. J Math Biol.

[45] Wang W, Zhao X-Q (2008). Threshold dynamics for compartmental epidemic models in periodic environments. J Dyn Differ Equ.

[46] Wolfram Mathematica (2012). Random number generation.

