# Supplementary material for: Optimal Seasonal Timing of Oral Azithromycin for Malaria
Source: Am J Trop Med Hyg. 2014 Nov 5;91(5):936–42. doi: 10.4269/ajtmh.13-0474 (PMC4228890; doi:10.4269/ajtmh.13-0474)
Supplement: Supplementary file 1 [file SD3.pdf]

Supplement to "Optimal seasonal timing of oral azithromycin for malaria"

Daozhou Gao[1], Abdou Amza[2], Baidou Nassirou[2], Boubacar Kadri[2], Nicholas Sippl-Swezey[1], Fengchen Liu[1], Sarah F. Ackley[1], Thomas M. Lietman[1,3,4] and Travis C. Porco[1,3*]

1 F.I. Proctor Foundation for Ophthalmological Research, University of California, San Francisco, California
2 Université Abdou Moumouni de Niamey, Programme National de Lutte Contre la Cécité, Niamey, Niger
3 Department of Ophthalmology, Department of Epidemiology and Biostatistics, University of California, San Francisco, California
4 Institute for Global Health, University of California, San Francisco, California

1. Varying the cutoff value for the high/low abundance season

A cutoff value is used to define the high abundance season, so that the high abundance season is defined as the time of year when the mosquito abundance exceeds some fraction (say 5% or 10%) of the peak abundance. For a given parameter set, the values of the cumulative hazard of infection (CHI) and entomological inoculation rate (EIR) are strictly increasing in the duration of the high mosquito abundance season. When we choose a larger cutoff value (such as 10% instead of 5%), we are thereby defining the high mosquito abundance season to be shorter. The two measures CHI and EIR are irrelevant to the treatment times, because they are derived from the model without treatment (please refer to their definitions in the Appendix).

In the following table, regions 1, 2, 3, 4 denote respectively the time during which mosquito abundance is greater than the threshold used to define the peak abundance (e.g. 5%, 10%) of the peak value (the peak transmission season), one month prior to this peak season, one month following this peak season, and finally, the rest of the year. In this table, we compared the CHI (evaluated over the peak abundance season for mosquitoes) between two groups of scenarios: (1) those for which the optimal treatment time was during the high season itself, and (2) those for which the optimal treatment time was during the low season. We report the ratio of these in the second column (Comparison ratio for CHI), and we found that the average CHI and EIR for scenarios yielding an optimal time in the high abundance season are still lower than those in the low abundance season for different thresholds ranging from 3% to 15%. For the base case scenario from the main text (bold), the CHI was over 23 times higher for scenarios with an optimal time in the low season than it was for scenarios with an optimal time in the high season. Similarly, we computed a comparison ratio for the EIR over the peak season between scenarios where the optimal time was in the high season and where the optimal time

was in the low season (column 3, Comparison ratio for EIR). The last column contains the number of scenarios where their optimal treatment times lie in regions 1,2,3,4, respectively.

Cutoff value	Comparison ratio for CHI (see text)	Comparison ratio for EIR (see text)	Number of scenarios in regions 1, 2, 3, 4
3%	1:7.4	1:5.0	{135,16, 2357, 3064}
**5%**	**1:23.3**	**1:16.5**	**{30,10, 1365,4167}**
8%	1:26.1	1:18.3	{27,11, 548,4986}
10%	1:26.6	1:18.3	{23,14, 286,5249}
12%	1:23.0	1:15.0	{15,22, 131,5404}
15%	1:24.4	1:15.7	{13,23, 30,5506}

2. The algorithm for finding the optimal time for mass drug administration (MDA)

For a given parameter set and initial treatment time $\tau_1 = 0, 0.01, 0.02, \cdots, 0.99, 1.0$, we obtain a stable periodic solution by solving the impulsive differential equations until the Euclidean distance of solution at times τ_i and τ_{i+1} is less than 10^{-7}. Then we calculate the annual prevalence, $P(\tau_1)$, and the total annual incidence of infection, $Q(\tau_1)$, from time τ_{i+1} to $\tau_{i+2} = \tau_{i+1} + 1$. Finally, we find the times that correspond to the minimal $P(\tau_1)$ and $Q(\tau_1)$, respectively, which are defined as the optimal treatment times in terms of annual prevalence and the total incidence, respectively, for that parameter set. If the minimal annual prevalence is less than 10^{-5} for some treatment time, then extinction effectively occurs, and the corresponding parameter set is discarded. For each qualified simulation (where the disease is still persistent after treatment any time) we find that there always exists a unique optimal treatment time.

3. Using a high-efficacy antimalarial drug

Our analytical framework can be used to determine the optimal seasonal timing of any antimalarial drug. Our analysis was restricted to azithromycin, due to the availability of data from an azithromycin treatment study, the Partnership for the Rapid Elimination of Trachoma study, Niger arm. For mass treatment with a high-efficacy antimalarial drug, our conclusions remain unchanged. For example, if we used the same parameter ranges except that of the curative efficacy which is changed from [0.4,0.8] to [0.75,0.95], then we obtain a smoothed

probability density plot (Figure 3) of optimal treatment times with respect to annual prevalence (solid line) and incidence (dashed line), respectively, which is almost the same as Figure 2a.

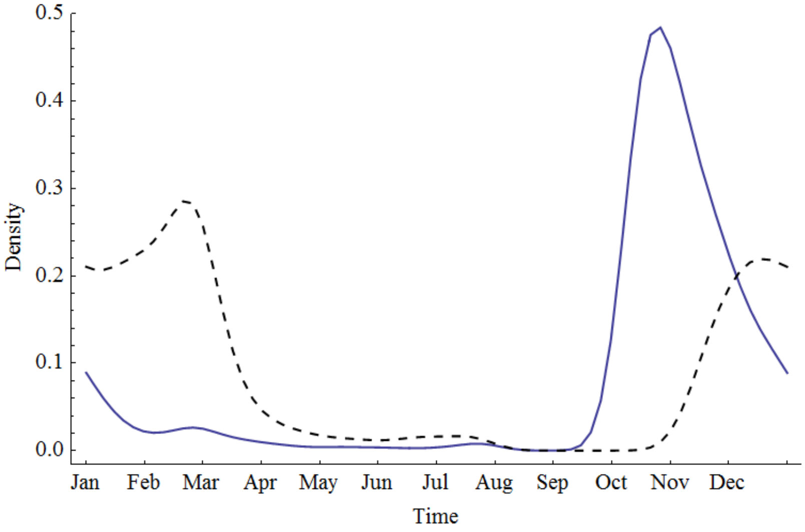

Figure 3

4. The distribution of the timing of mass drug administration which could lead to elimination

For the discarded simulations (4428) (page 3, main text), malaria is mostly eliminated even without treatment (4194) or with treatment at any time (111). However, there are a small number (123) of simulations where the disease dies out if the treatment is conducted at certain times, although it persists if treatment is conducted at some other times. For instance, if the treatment time is optimal or sufficiently close to optimal, elimination could result, but a poor choice of mass treatment time would not be sufficient for elimination. The following smoothed probability density plot (see Figure 4a) gives an insight of the distribution of the timing of drug distribution that leads to elimination for the 123 simulations. It also implies that the best treatment time is at low abundance season or after high abundance season.

From Table 2 in the text, the PRCC of the difference in the annual person-time of infection, comparing best and worst with respect to p and q_i, are 0.84 and 0.66, respectively. The CLPRCC for the optimal treatment time with respect to p and q_i are only 0.04 and 0.01, respectively. Different values of p and q_i (coverage and efficacy) could have a large effect on prevalence, but have little effect on optimal timing.

Changes in treatment efficacy p and coverage rate q_i could result in a change of outcome from disease extinction to persistence or from disease persistence to extinction, and generate a different number of simulations where the outcome depends on the treatment time.

The model yields a relatively small number of scenarios of low transmission potential (low R_0) and high coverage and/or efficacy, where mass treatment could tip the balance against malaria, resulting in an overall $R_0<1$ and elimination. Of course, the manuscript does not address the ability of MDA to eliminate malaria.

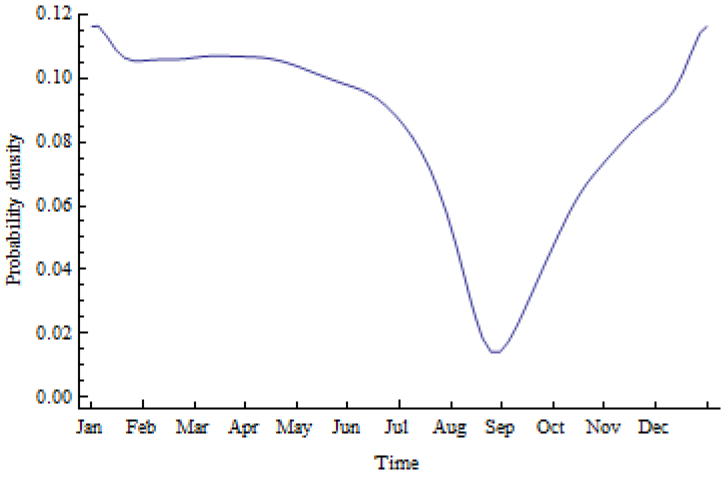

Figure 4a)

We found that the choices of p and q_i may make little difference when other parameters remain the same values in the 123 simulations we mentioned above (those for which the outcome depends on the treatment time). Specifically, we conducted three analyses as follows.

- Beginning with the original parameter sets, we chose other values as follows. First, we divided the parameter ranges of p and q_i into 10 equal subintervals, respectively, forming an equally spaced grid (an 11×11 array of (p, q_i)). Then we simulated each parameter set with the 121 pairs of (p, q_i) and obtained a similar distribution of the timing of drug distribution that leads to elimination (see Figure 4b).

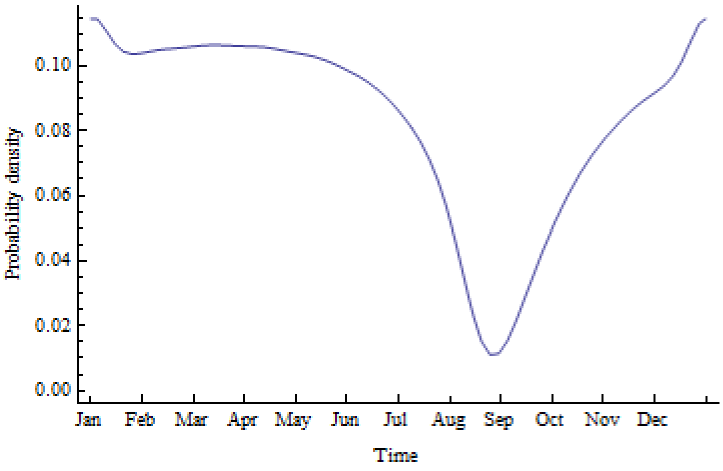

Figure 4b)

- In addition, we conducted 100,000 simulations with the same parameter ranges as the original 10,000 simulations (in the original manuscript), and obtained 1,178 simulations where the disease dies out at some treatment time while persists at the remaining treatment time. A similar timing distribution is obtained for these as well (see Figure 4c).

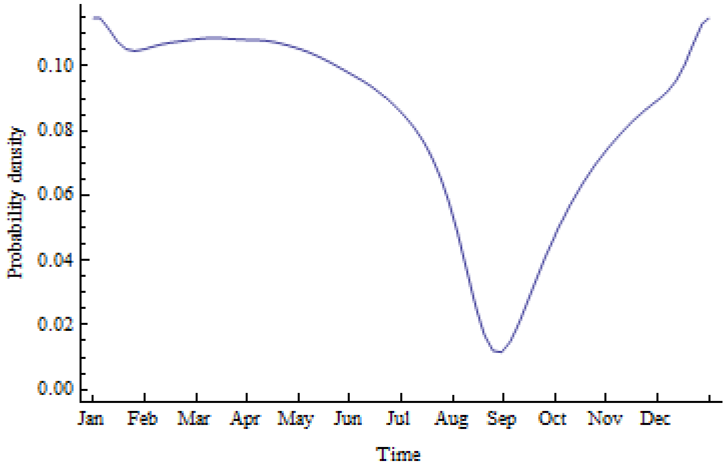

Figure 4c)

- We also conducted an additional 10,000 simulations in which the parameter ranges of p and q_i were enlarged; specifically, from [0.4,0.8] and [0.6,0.9] to [0.1,0.8] and [0.5,1], respectively. We obtained 88 simulations where the disease dies out at some treatment time while persists at the remaining treatment time. The timing distribution of these 88 simulations (see Figure 4d) is essentially the same as above.

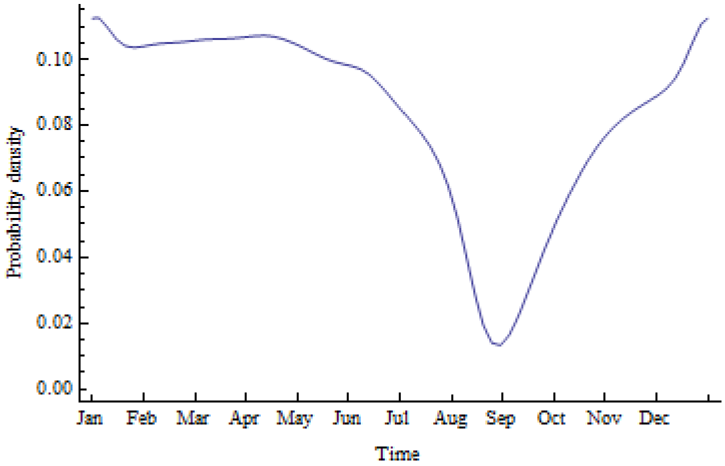

Figure 4d)

Note that elimination by azithromycin is not a common occurrence in our model, and we do not imply that mass administration of azithromycin is expected to lead to elimination.

5. Extended model equations, parameter ranges and additional simulation results.

The extrinsic incubation period in mosquitoes, denoted by 1/η, is a potentially important factor in modeling malaria transmission. There are several ways to incorporate its role, namely, introducing an exposed class for mosquitoes, timing infected mosquitoes by a constant exp(-μ/η), or developing a delay differential equations model (see for example AronMay-Book1982, SmithMcKenzie-MJ2004, RuanXiaoBeier-BMB2008, Chitnis et al-BMB2008, Cosner et al-JTB2009, LouZhao-JMB2011, Smith et al-PLoSPat2012). We conducted numerical simulations for the first two ways and found that there is no significant difference in the distribution of optimal treatment time when compared with our original model. A possible reason is that if there are incubation period delays, then large numbers of transmission cycles do not happen during the high abundance season and less transmission favors earlier treatment. However, as expected, malaria goes extinct in more parameter sets because a large proportion of mosquitoes die before they develop infectiousness.

Another omission in our simple model is the contribution of adult humans. In a sensitivity analysis, we included adults and proposed an age-structured malaria transmission model. While this affects the distribution of the optimal treatment times, it does not qualitatively change the central tendency of them, which still occurs in the low abundance season or after high abundance season.

In what follows, we use *h* for infected humans, *v* for infected mosquitoes, v_e for exposed mosquitoes, v_i for infectious mosquitoes, s_1/h_1 for susceptible/infected children, s_2/h_2 for susceptible/infected adults. *H* (or *H(t)* in Case II) and *V(t)* are the total number of humans and mosquitoes, respectively. Similar to Figure 2a, we made four smoothed probability density plot (Figures 5a-5d) of optimal treatment times with respect to annual prevalence (solid line) and incidence (dashed line), for Case I, I', II, and II', respectively.

Case I: SEI pattern for mosquitoes. 1/η-the extrinsic incubation period in mosquitoes

$$\frac{dh(t)}{dt} = ab\frac{\sigma H - h(t)}{H}v_i(t) - rh(t) - p\sum_{i=1}^{\infty} q_i h(\tau_i^-)\delta(t-\tau_i),$$

$$\frac{dv_e(t)}{dt} = ac\frac{h(t)}{H}(V(t) - v_e(t) - v_i(t)) - \eta v_e(t) - \mu v_e(t),$$

$$\frac{dv_i(t)}{dt} = \eta v_e(t) - \mu v_i(t).$$

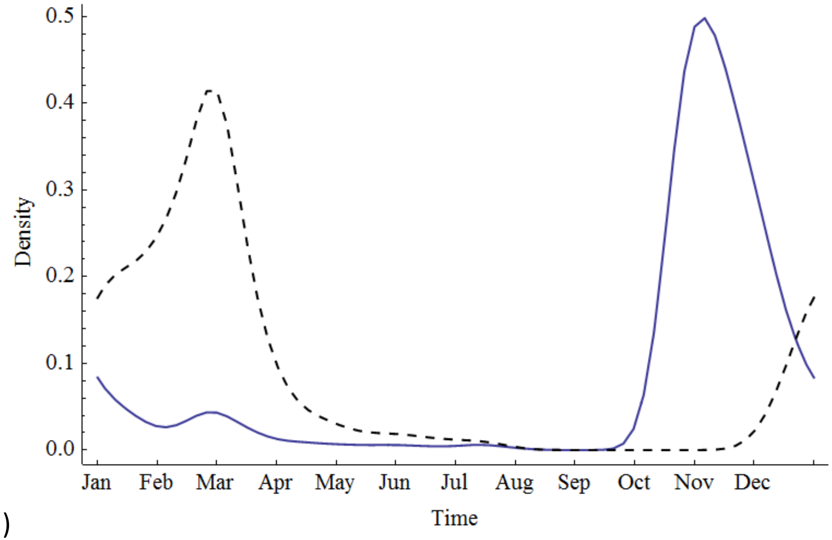

Figure 5a)

Case I': Incorporate mosquito mortality during the latent period but ignoring the delay for pathogen latency in mosquitoes (SmithMcKenzie-MJ2004)

$$\frac{dh(t)}{dt} = abe^{-\mu/\eta}\,\frac{\sigma H - h(t)}{H}v(t) - rh(t) - p\sum_{i=1}^{\infty} q_i h(\tau_i^-)\delta(t-\tau_i),$$

$$\frac{dv(t)}{dt} = ac\frac{h(t)}{H}(V(t) - v(t)) - \mu v(t).$$

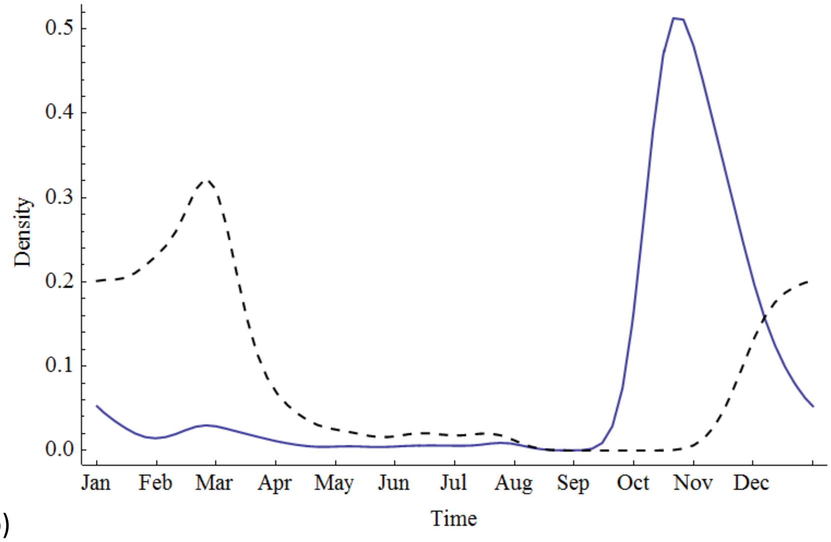

Figure 5b)

Here we adopted the idea of disease latency as in SmithMcKenzie-MJ2004, but used notation from Cosner et al-JTB2009 (see Equations (2.1-2.2) in this paper). Since the mosquito population size seasonally varies, we formulated our model in terms of population instead of proportions of population.

Specifically, in SmithMcKenzie-MJ2004, equations

$$\dot{X} = mabZ(1-X) - rX,$$
$$\dot{Z} = acX(e^{-gn} - Z) - gZ,$$

are in terms of **the proportion of infectious humans** (X) **and mosquitoes** (Z), respectively.

In Cosner et al-JTB2009, equations

$$\frac{dx}{dt} = Maby(1-x) - rx,$$
$$\frac{dy}{dt} = acx(e^{-\mu\tau} - y) - \mu y,$$

are in terms of **the proportion of infectious humans** (x) **and mosquitoes** (y), respectively. After a change of variables $X = xH$ and $e^{-\mu\tau}Y = yV$, they become

$$\frac{dX}{dt}\cdot\frac{1}{H} = \frac{V}{H}abe^{-\mu\tau}\frac{Y}{V}(1-\frac{X}{H}) - r\frac{X}{H},$$
$$\frac{dY}{dt}\cdot\frac{e^{-\mu\tau}}{V} = ac\frac{X}{H}(e^{-\mu\tau} - e^{-\mu\tau}\frac{Y}{V}) - \mu e^{-\mu\tau}\frac{Y}{V},$$

or

$$\frac{dX}{dt} = \frac{abe^{-\mu\tau}}{H}(H-X) - rX,$$
$$\frac{dY}{dt} = \frac{ac}{H}X(V-Y) - \mu Y,$$

where **X and Y are the numbers of infected humans and mosquitoes**, respectively. Note that in both SmithMcKenzie-MJ2004 and Cosner et al-JTB2009, they used n (or τ) to denote the incubation period in mosquitoes, while we used $1/\eta$.

Case I'': Delay differential equations (AronMay-Book1982)

$$\frac{dh(t)}{dt} = ab\frac{\sigma H - h(t)}{H}v_i(t) - rh(t) - p\sum_{i=1}^{\infty} q_i h(\tau_i^-)\delta(t-\tau_i),$$

$$\frac{dv_e(t)}{dt} = ac\frac{h(t)}{H}(V(t) - v_e(t) - v_i(t)) - ac\frac{h(t-1/\eta)}{H}(V(t-1/\eta) - v_e(t-1/\eta) - v_i(t-1/\eta))e^{-\mu/\eta} - \mu v_e(t),$$
$$\frac{dv_i(t)}{dt} = ac\frac{h(t-1/\eta)}{H}(V(t-1/\eta) - v_e(t-1/\eta) - v_i(t-1/\eta))e^{-\mu/\eta} - \mu v_i(t).$$

Case II: Consider the contribution of both children and adults

$$\frac{ds_1(t)}{dt} = -ab_1 \frac{s_1(t)}{H(t)} v_i(t) + r_1 h_1(t) + p\sum_{k=1}^{\infty} q_k h_1\left(\tau_k^-\right)\delta\left(t-\tau_k\right) - \alpha s_1(t) + \varepsilon - \mu_1 s_1(t),$$

$$\frac{dh_1(t)}{dt} = ab_1 \frac{s_1(t)}{H(t)} v_i(t) - r_1 h_1(t) - p\sum_{k=1}^{\infty} q_k h_1\left(\tau_k^-\right)\delta\left(t-\tau_k\right) - \alpha h_1(t) - \mu_1 h_1(t),$$

$$\frac{ds_2(t)}{dt} = -ab_2 \frac{s_2(t)}{H(t)} v_i(t) + r_2 h_2(t) + p\sum_{k=1}^{\infty} q_k' h_2\left(\tau_k^-\right)\delta\left(t-\tau_k\right) + \alpha s_1(t) - \mu_2 s_2(t),$$

$$\frac{dh_2(t)}{dt} = ab_2 \frac{s_2(t)}{H(t)} v_i(t) - r_2 h_2(t) - p\sum_{k=1}^{\infty} q_k' h_2\left(\tau_k^-\right)\delta\left(t-\tau_k\right) + \alpha h_1(t) - \mu_2 h_2(t),$$

$$\frac{dv_e(t)}{dt} = ac_1 \frac{h_1(t)}{H(t)}(V(t) - v_e(t) - v_i(t)) + ac_2 \frac{h_2(t)}{H(t)}(V(t) - v_e(t) - v_i(t)) - \eta v_e(t) - \mu v_e(t),$$

$$\frac{dv_i(t)}{dt} = \eta v_e(t) - \mu v_i(t), \qquad H(t) = s_1(t) + h_1(t) + s_2(t) + h_2(t).$$

Since the total number of children and adults satisfies

$$\frac{d(s_1 + h_1)}{dt} = \varepsilon - (\alpha + \mu_1)(s_1(t) + h_1(t)),$$

$$\frac{d(s_2 + h_2)}{dt} = \alpha(s_1(t) + h_1(t)) - \mu_2(s_2(t) + h_2(t)),$$

respectively, we have $s_1(t) + h_1(t) \to \varepsilon/(\alpha + \mu_1)$ and $s_2(t) + h_2(t) \to \varepsilon/(\alpha + \mu_1)\cdot \alpha/\mu_2$ as $t \to \infty$.

Let $H = \lim_{t\to\infty} H(t) = \left(1 + \frac{\alpha}{\mu_2}\right)\frac{\varepsilon}{\alpha + \mu_1}$ and $\sigma = \frac{\mu_2}{\alpha + \mu_2}$. We obtain a reduced equivalent system

$$\frac{dh_1(t)}{dt} = ab_1 \frac{\sigma H - h_1(t)}{H} v_i(t) - r_1 h_1(t) - p\sum_{k=1}^{\infty} q_k h_1\left(\tau_k^-\right)\delta\left(t-\tau_k\right) - \alpha h_1(t) - \mu_1 h_1(t),$$

$$\frac{dh_2(t)}{dt} = ab_2 \frac{(1-\sigma)H - h_2(t)}{H} v_i(t) - r_2 h_2(t) - p\sum_{k=1}^{\infty} q_k' h_2\left(\tau_k^-\right)\delta\left(t-\tau_k\right) + \alpha h_1(t) - \mu_2 h_2(t),$$

$$\frac{dv_e(t)}{dt} = ac_1 \frac{h_1(t)}{H}(V(t) - v_e(t) - v_i(t)) + ac_2 \frac{h_2(t)}{H}(V(t) - v_e(t) - v_i(t)) - \eta v_e(t) - \mu v_e(t),$$

$$\frac{dv_i(t)}{dt} = \eta v_e(t) - \mu v_i(t).$$

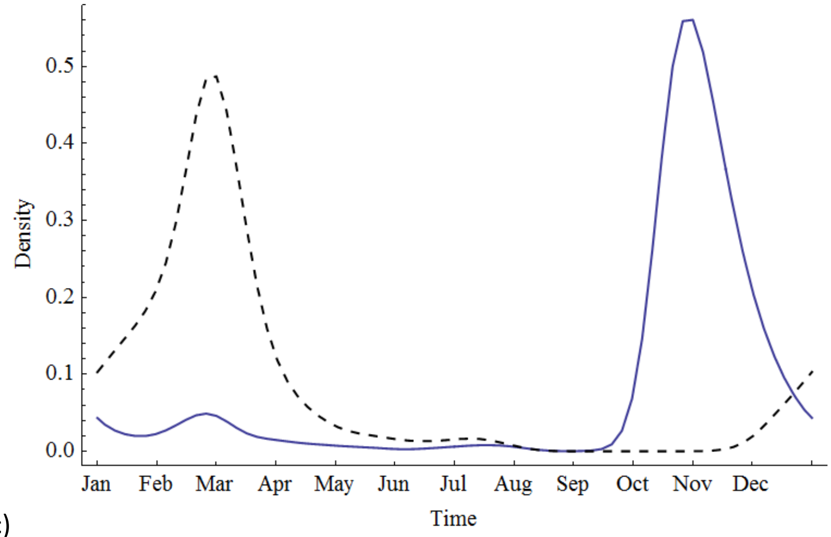

Figure 5c)

Case II': Incorporate mosquito mortality during the latent period but ignoring the delay for pathogen latency in mosquitoes (SmithMcKenzie-MJ2004)

$$\frac{dh_1(t)}{dt} = ab_1 e^{-\mu/\eta} \frac{\sigma H - h_1(t)}{H} v(t) - r_1 h_1(t) - p\sum_{k=1}^{\infty} q_k h_1(\tau_k^-)\delta(t-\tau_k) - \alpha h_1(t) - \mu_1 h_1(t),$$

$$\frac{dh_2(t)}{dt} = ab_2 e^{-\mu/\eta} \frac{(1-\sigma)H - h_2(t)}{H} v(t) - r_2 h_2(t) - p\sum_{k=1}^{\infty} q_k' h_2(\tau_k^-)\delta(t-\tau_k) + \alpha h_1(t) - \mu_2 h_2(t),$$

$$\frac{dv(t)}{dt} = ac_1 \frac{h_1(t)}{H}(V(t) - v(t)) + ac_2 \frac{h_2(t)}{H}(V(t) - v(t)) - \mu v(t),$$

where

$$H = \left(1 + \frac{\alpha}{\mu_2}\right)\frac{\varepsilon}{\alpha + \mu_1}, \quad \sigma = \frac{\mu_2}{\alpha + \mu_2}.$$

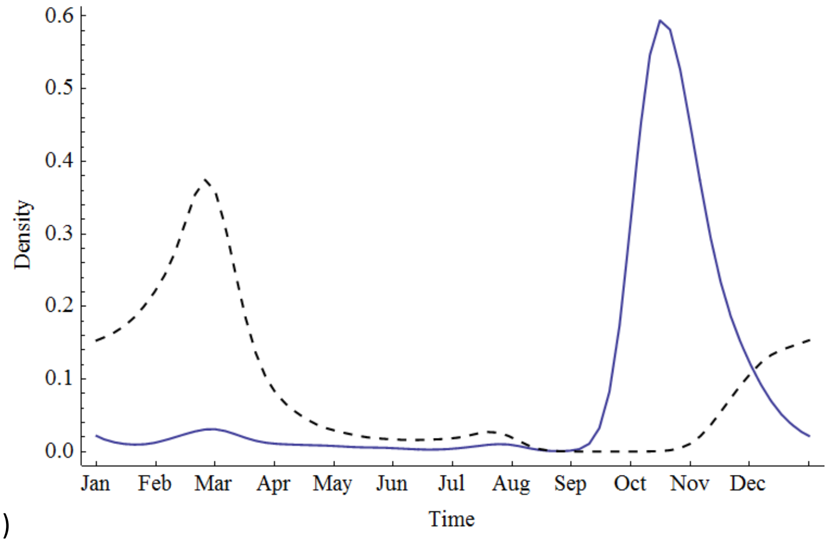

Figure 5d)

Case II'': Delay differential equations (AronMay-Book1982)

$$\frac{dh_1(t)}{dt} = ab_1 \frac{\sigma H - h_1(t)}{H} v_i(t) - r_1 h_1(t) - p\sum_{k=1}^{\infty} q_k h_1(\tau_k^-)\delta(t-\tau_k) - \alpha h_1(t) - \mu_1 h_1(t),$$

$$\frac{dh_2(t)}{dt} = ab_2 \frac{(1-\sigma)H - h_2(t)}{H} v_i(t) - r_2 h_2(t) - p\sum_{k=1}^{\infty} q'_k h_2(\tau_k^-)\delta(t-\tau_k) + \alpha h_1(t) - \mu_2 h_2(t),$$

$$\begin{aligned}\frac{dv_e(t)}{dt} &= ac_1 \frac{h_1(t)}{H}(V(t)-v_e(t)-v_i(t)) + ac_2 \frac{h_2(t)}{H}(V(t)-v_e(t)-v_i(t)) \\ &\quad - ac_1 \frac{h_1(t-1/\eta)}{H}(V(t-1/\eta)-v_e(t-1/\eta)-v_i(t-1/\eta))e^{-\mu/\eta} \\ &\quad - ac_2 \frac{h_2(t-1/\eta)}{H}(V(t-1/\eta)-v_e(t-1/\eta)-v_i(t-1/\eta))e^{-\mu/\eta} - \mu v_e(t),\end{aligned}$$

$$\begin{aligned}\frac{dv_i(t)}{dt} &= ac_1 \frac{h_1(t-1/\eta)}{H}(V(t-1/\eta)-v_e(t-1/\eta)-v_i(t-1/\eta))e^{-\mu/\eta} \\ &\quad + ac_2 \frac{h_2(t-1/\eta)}{H}(V(t-1/\eta)-v_e(t-1/\eta)-v_i(t-1/\eta))e^{-\mu/\eta} - \mu v_i(t),\end{aligned}$$

where

$$H = \left(1+\frac{\alpha}{\mu_2}\right)\frac{\varepsilon}{\alpha+\mu_1}, \quad \sigma = \frac{\mu_2}{\alpha+\mu_2}.$$

Table 1: Parameters of the malaria model with description, range, baseline, unit and reference for Case I and I'.

	Description	Range	Baseline	Unit	References
a	the number of bites per mosquito per month	3-30	3-15	bites per mosquito per month	[18, 22, 23, 24]
b	transmission probability from infected mosquitoes to susceptible children per bite	0.01-0.8	0.1-0.5	per bite	[18, 22, 25, 26, 27]
c	transmission probability from infected children to susceptible mosquitoes per bite	0.072-0.64	0.1-0.5	per bite	[23, 25, 26, 27]
$1/r$	duration of infectiousness	0.7-10	0.7-3	month	[18, 24, 25, 26, 27]
$1/\mu$	lifespan of mosquitoes	0.2-36	0.25-1	month	[23, 24, 25]
σ	the proportion of people under 12	0-1	1/4-1/2	--	[28]
H	number of humans	50-1000	250-600	--	Assume
m	average ratio of mosquitoes to humans	1-10	1-4	mosquitoes per human	[18, 22, 23, 24]
k	measure of the duration of high season	0-1	0.984-0.997	--	[20]
p	curative efficacy of single dose of azithromycin	0-1	0.4-0.8	--	Assume
q_i	treatment coverage	0-1	0.6-0.9	--	Assume
τ_1	initial mass administration time	0-1	0-1	year	Assume
$1/\eta$	the extrinsic incubation period in mosquitoes	0.1-1.1	0.16-0.5	month	[18, 24, 25, 26, 27]

Table 2: Parameters of the malaria model with description, range, baseline, unit and reference for Case II and II'.

	Description	Range	Baseline	Unit	References
a	the number of bites per mosquito per month	3-30	3-15	bites per mosquito per month	[18, 22, 23, 24]
b_1	transmission probability from infected mosquitoes to susceptible children per bite	0.01-0.8	0.1-0.5	per bite	[18, 22, 25, 26, 27]
b_2	transmission probability from infected mosquitoes to susceptible adults per bite	0.01-0.8	0-0.3	per bite	[18, 22, 25, 26, 27]
c_1	transmission probability from infected children to susceptible mosquitoes per bite	0.072-0.64	0.1-0.5	per bite	[23, 25, 26, 27]
c_2	transmission probability from infected adults to susceptible mosquitoes per bite	0.072-0.64	0-0.3	per bite	[23, 25, 26, 27]
$1/r_1$	duration of infectiousness in children	0.7-10	0.7-3	month	[18, 24, 25, 26, 27]
$1/r_2$	duration of infectiousness in adults	0.5-10	0.5-1.5	month	[18, 24, 25, 26, 27]
$1/\mu_1$	life expectancy of children	480-800	480-800	month	Assume
$1/\mu_2$	life expectancy of adults	240-400	240-400	month	Assume
$1/\mu$	lifespan of mosquitoes	0.2-36	0.25-1	month	[23, 24, 25]
m	average ratio of mosquitoes to humans	1-10	1-4	mosquitoes per human	[18, 22, 23, 24]
k	measure of the duration of high season	0-1	0.984-0.997	--	[20]
p	curative efficacy of single dose of azithromycin	0-1	0.4-0.8	--	Assume
q_i	treatment coverage of children	0-1	0.6-0.9	--	Assume
$q_i^{'}$	treatment coverage of adults	0-1	0.6-0.9	--	Assume
τ_1	initial mass administration time	0-1	0-1	year	Assume
$1/\eta$	the extrinsic incubation period in mosquitoes	0.1-1.1	0.16-0.5	month	[18, 24, 25, 26, 27]

References:

[SmithMcKenzie-MJ2004] Smith DL, McKenzie FE (2004) Statics and dynamics of malaria infection in Anopheles mosquitoes. Malar J 3: 13.

[AronMay-Book1982] Aron JL, May RM (1982) The population dynamics of malaria. In: Anderson RM, ed. Population dynamics and infectious disease. London: Chapman and Hall. pp 139–179.

[RuanXiaoBeier-BMB2008] Ruan S, Xiao D, Beier JC (2008) On the delayed Ross-Macdonald model for malaria transmission. Bulletin of Mathematical Biology Vol. 70, pp 1098-1114.

[Cosner et al-JTB2009] Cosner C, Beier JC, Cantrell RS, Impoinvil D, Kapitanski L, Potts MD, Troyo A, Ruan S (2009) The effects of human movement on the persistence of vector borne diseases, Journal of Theoretical Biology Vol. 258, pp 550-560.

[Chitnis et al-BMB2008] Chitnis N, Hyman JM, Cushing JM (2008) Determining important parameters in the spread of malaria through the sensitivity analysis of a mathematical model. Bulletin of Mathematical Biology Vol. 70, pp 1272-1296.

[LouZhao-JMB2011] Lou Y, Zhao XQ (2011) A reaction–diffusion malaria model with incubation period in the vector population. Journal of Mathematical Biology Vol. 62, pp 543-568.

[Smith et al-PLoSPat2012] Smith DL, Battle KE, Hay SI, Barker CM, Scott TW, McKenzie FE (2012) Ross, Macdonald, and a theory for the dynamics and control of mosquito-transmitted pathogens. PLoS Pathogens. 8(4): e1002588.
